# BeadArray Expression Analysis Using Bioconductor

**DOI:** 10.1371/journal.pcbi.1002276

**Published:** 2011-12-01

**Authors:** Matthew E. Ritchie, Mark J. Dunning, Mike L. Smith, Wei Shi, Andy G. Lynch

**Affiliations:** 1 Bioinformatics Division, The Walter and Eliza Hall Institute of Medical Research, Parkville, Victoria, Australia; 2 Department of Medical Biology, The University of Melbourne, Parkville, Victoria, Australia; 3 Cancer Research UK, Cambridge Research Institute, Cambridge, United Kingdom; 4 Department of Oncology, University of Cambridge, Cambridge, United Kingdom; 5 Department of Computer Science and Software Engineering, The University of Melbourne, Parkville, Victoria, Australia; Whitehead Institute, United States of America

## Abstract

Illumina whole-genome expression BeadArrays are a popular choice in gene profiling studies. Aside from the vendor-provided software tools for analyzing BeadArray expression data (GenomeStudio/BeadStudio), there exists a comprehensive set of open-source analysis tools in the Bioconductor project, many of which have been tailored to exploit the unique properties of this platform. In this article, we explore a number of these software packages and demonstrate how to perform a complete analysis of BeadArray data in various formats. The key steps of importing data, performing quality assessments, preprocessing, and annotation in the common setting of assessing differential expression in designed experiments will be covered.

## Introduction

Microarrays are a standard laboratory technique for high-throughput gene expression profiling in genomics research. The BeadArray microarray platform from Illumina Inc. (San Diego, CA) consists of an array of randomly packed beads, each bead bearing many copies of a particular 50-mer oligonucleotide sequence (the “probe”). Each BeadArray contains a collection of probes designed to interrogate the majority of protein-coding transcripts in a given organism (human, mouse, or rat) along with a large set of both positive and negative control probes. Due to the random sampling of beads during the manufacturing process, the number and arrangement of replicate beads varies from array to array.

**Figure 1:**
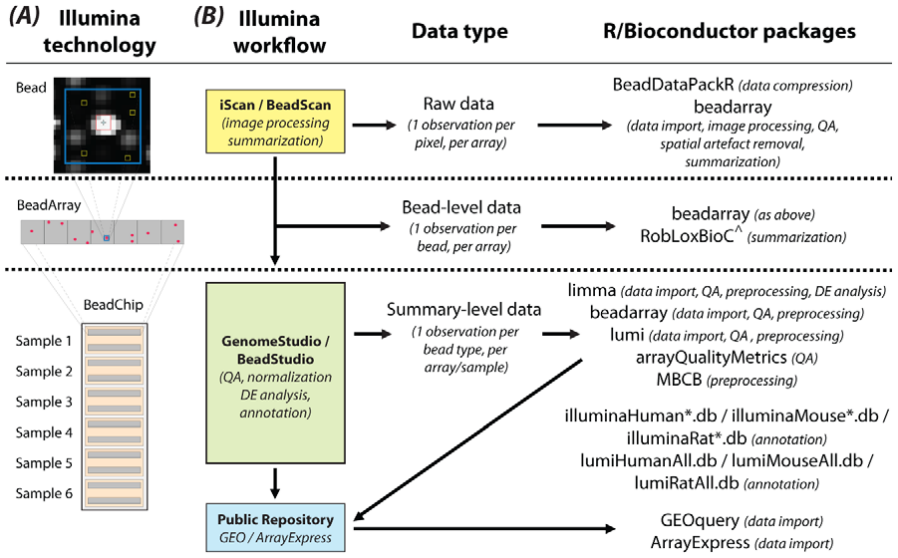
Overview of the technology and workflow. (A) A zoomed view of a typical bead (top) with the pixels that contribute to the overall (red square) and local background (yellow squares) signals marked. Many replicate beads that contain the same 50-mer oligo are located on each BeadArray (middle) to ensure robust measures of expression can be obtained for each probe in a given sample. Around 48,000 different probe types are assayed in this way per sample. These BeadArrays come from a WG-6 BeadChip (bottom), which is made up of a total of 12 arrays, which are paired to allow transcript abundance to be measured in a total of six samples per BeadChip. (B) Summarizes the various data formats available along with the Illumina workflow associated with the different levels of data. Data can be in raw form, where pixel-level data are available from TIFF images, allowing the complete data processing pipeline, including image analysis, to be carried out in R. The next level, referred to as bead-level, refers to the availability of intensity and location information for individual beads. In this format, a given probe will have a variable number of replicate intensities per sample. Processed data, where replicate intensities have been summarized and outliers removed to give a mean, a measure of variability, and a number of observations per probe in each sample, is the most commonly available format. Summary data are usually obtained directly from Illumina's BeadStudio/GenomeStudio software, but can also be retrieved from public repositories such as GEO or ArrayExpress. The right-hand column of this figure indicates the R/Bioconductor packages that can handle data in these different formats. Probe annotation packages are also listed. List of abbreviations and footnotes used in this figure: QA, quality assessment; DE, differential expression; ∧, package available from CRAN [46]; *, denotes chip-specific part of package name that depends upon platform version (e.g., v1, v2, v3, v4).

Multiple BeadArrays are grouped together to form a BeadChip, with gene expression products configured to have six (WG-6), eight (Ref-8), or 12 (HT-12) samples per chip. This format allows samples to be processed in parallel with benefits for experimental design, a key factor in the experimental workflow [1]. The hierarchy of data, from individual pixels that make up beads on a BeadArray for a WG-6 BeadChip, is illustrated in Figure 1A.

The experimental process for measuring transcript levels in a sample of interest involves labelling RNA and hybridizing this material to the probes on a BeadArray. The scanned intensities from these probes provide a snapshot of transcript abundance in a particular sample. Comparing the intensities obtained from different RNA species can provide researchers with insight into the molecular pathways regulating the system under investigation. There is a rich literature on the analysis of gene expression microarrays (see Smyth et al. 2003 [2], Allison et al. 2006 [3], or Reimers 2010 [4] for reviews), and while the main steps of an analysis such as quality assessment and normalization still apply, BeadArray data present a number of unique opportunities that may not be fully exploited by standard microarray analysis workflows. These include a high and variable level of intra-array replication of probes and a large set of negative controls. Specialized algorithms that make use of these features have been developed for Illumina BeadArrays to improve the results obtained from this technology.

The aim of this article is to provide a how-to guide for Illumina expression analysis, using packages from the open-source Bioconductor project [5]. The overall workflow of an Illumina analysis is summarized in Figure 1B. Analyses may begin with data at one of four starting points: raw data including the scanned TIFF images, bead-level data without the TIFF images, summarized output from BeadStudio/GenomeStudio, or data obtained from a public repository. Depending on the format available, different open-source tools from Bioconductor may be used to import and analyze the data (Figure 1B). The methods we routinely use in our own analyses of Illumina gene expression data are summarized in Table 1.

**Table 1:** Summary of the processing methods recommended for different levels of data.

Data Type	Analysis Task	Recommended Approach
All levels	Quality assessment	Examine scanner metrics
Raw^a^	Local background adjustment	Median background subtraction
Raw	Transformation	log_2_
Bead-level^b^	Spatial artefact detection & removal	BASH
Bead-level	Quality assessment	Examine image plots & boxplots
Bead-level	Summarization	Default Illumina method
Summary-level^c^	Data export from BeadStudio/ GenomeStudio	Non background corrected, non normalized, Sample and Control “Probe Profile” tables
Summary-level	Quality assessment	Examine boxplots of regular & control probes, MDS plots
Summary-level	Background correction	Normal-exponential convolution using negative controls
Summary-level	Normalization	Quantile
Summary-level	Transformation	log_2_
Summary-level	Estimation of proportion of expressed probes in a sample	Mixture model that uses negative controls (propexpr [29])
Summary-level	Probe filtering	Based on annotation quality
Summary-level	Differential expression analysis	Linear modelling using weights

a Raw data comprises one observation per pixel, per array.

b Bead-level data comprises one observation per bead, per array.

c Summary-level data comprises one observation per probe type, per sample.

The companion Bioconductor package *BeadArrayUseCases* [6] provides a vignette with a series of examples aimed at computational biologists wanting instruction on the specific commands involved in analyses from any starting level of data. Three experiments using three generations of BeadArray allow us to span the range of data levels and illustrate the use of specific functions from the *beadarray*, *limma*, and *GEOquery* packages. We also demonstrate how to extract information from chip-specific annotation packages.

## Choosing a Starting Point for the Analysis of BeadArray Data

The first decision facing the bioinformatician may be what data to use as the starting point for their analysis. If all primary data formats (raw data including TIFFs, bead-level data without TIFFs, or summarized data) have been made available, then it should be clear that starting from the TIFF images gives the greatest amount of control over the steps being performed at each stage. In most situations, the default processing methods employed by Illumina to extract intensities from the TIFFs and summarize these values within each sample produce good intensity estimates. Whether these processing steps are carried out in R (see vignette), or using vendor-provided software, is obviously up to the user; however, the ability to perform the entire analysis in a platform-independent, reproducible, and flexible manner in R will be appealing to many computational biologists. In addition, image registration issues [7] and spatial artefacts [8] can only be managed if raw or bead-level data are available. While the impact of such events can range from mild to catastrophic, if an analysis begins with summarized data, then the user will only see the symptoms of such errors, and be unable to deal with the potential cause of the problem.

## Quality Assessment for All Levels of Data

**Figure 2:**
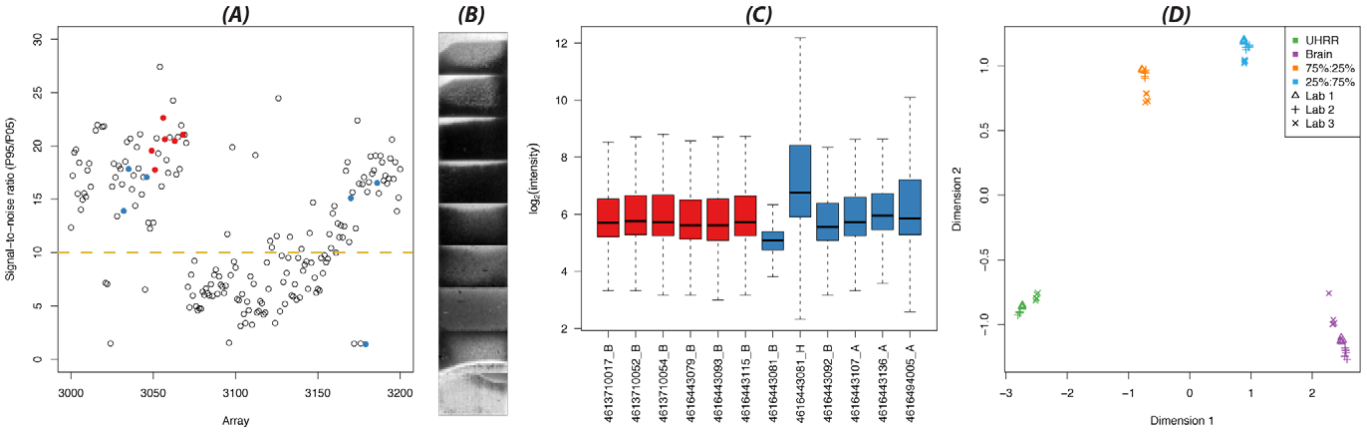
Various diagnostic plots which are useful for quality assessment. Where scanner metrics information is available, arrays within a particular experiment can be compared to each other, or to a wider set from the same core facility. In (A), a per array signal-to-noise value (95th percentile of signal divided by the 5th percentile) is plotted for 200 consecutive BeadArrays, with the arrays from the experiment in question highlighted in color (blue or red). Low signal-to-noise ratios indicate a poor dynamic range of intensities and can highlight problems with array processing when they occur sequentially over time. At the individual array level, sub-array artefacts can be detected using spatial plots of the intensities across the BeadArray surface (B) and removed using BASH and outlier removal. For a between sample display, boxplots of the intensities from different arrays within an experiment can highlight samples with unusual signal distributions (C). The relationships between different samples can also be assessed using a multi-dimensional scaling (MDS) plot (D), which can highlight true biological differences between samples (in this example, the difference between UHRR and Brain in dimension 1 and the pure versus mixed samples in dimension 2), as well as technical effects due to lab, experiment date, etc., which may also need to be accounted for in the modelling.

Irrespective of whether raw, bead-level, or summarized data are being analyzed, the first opportunity to assess the quality of an experiment occurs as the arrays are being scanned, and without the need for specialized software. The scanner produces a text file that contains various signal-based array quality measures. As an example, Figure 2A shows the signal-to-noise ratio (SNR) for 200 arrays, including the 12 arrays from the first data set analyzed in the vignette. Of these 12 samples, one has a very low SNR, which warrants further investigation and provides grounds for down-weighting or removal of this sample from the analysis. The value and interpretation of these metrics will be influenced by many factors, so it is advisable for laboratories to keep an historical record of these values to assist in the detection of systematic problems during processing and in the identification of outlier samples.

## Raw and Bead-Level Data Analysis

To obtain raw or bead-level data, modifications to the default scanning settings in BeadScan or iScan are required. Currently, the *beadarray* package [9] is the only Bioconductor package that can process these raw data either in the form obtained from the scanner or in a compact representation via the *BeadDataPackR* package [10].

The import of raw data is handled using the readIllumina function. The availability of TIFF images depends on scanner settings (jpegs are provided by default), and where present *beadarray* can extract background, foreground, and total intensities to the user's specifications. In particular, using a more robust measure of the local background intensity (median) has been shown to be beneficial [7]. If TIFF images are not available, then the user begins with Illumina's foreground intensities, calculated by subtraction of the local background measure from the total intensity.

As with other microarrays, it is usual to analyze data on the 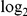 scale, and therefore the plotting and analysis methods used in *beadarray* employ this transformation by default. The first within-sample quality plots that one can produce are overall image plots of the array surface (Figure 2B) to look for obvious spatial problems. In addition, the checkRegistration function provides a convenient way to assess whether the reported bead centers agree with the bead locations in the raw images.

Although Illumina's processing steps include the removal of outliers for each bead type, we find that this is not sufficient to account for all spatial artefacts that may occur on the array surface. Although it is a computationally expensive operation, we routinely use the BASH tool in *beadarray* to detect and remove spatial artefacts [8], [11]. This method is based upon the principles of the *Harshlight* [12] package for Affymetrix, but works on a within-array basis rather than between arrays, using the within-array replication to generate similar performance. The use of BASH is recommended, but the parameters may need to be tuned to achieve good performance between different labs or experiments.

Other useful diagnostic plots such as boxplots can be used to reveal unusual signal distributions (Figure 2C) and plots of control probes (positive or negative) can highlight processing problems that may warrant sample removal. For convenience, the expressionQCPipeline function automatically generates all recommended quality control plots for a given data set.

After image processing, a key step is the reduction of raw data (many values per probe type) to summary data (one value per probe type) in order that we might apply the methods detailed in the next section. *beadarray* offers flexibility in the definition of which beads to include in summarization and the choice of summary statistic and transformation applied to the raw data (the key aspects of summarization). The standard summary statistics to use in *beadarray* are the mean and standard error of the log-intensity (Illumina's standard statistics to report are the mean and standard error of the raw intensity). Note that the standard error is important for Illumina BeadArrays, as the random design means that we will have differing levels of confidence from one measure of intensity to the next. Besides variations of the standard Illumina outlier removal method offered by *beadarray*, other robust summary options are possible as described in Kohl and Deigner (2010) [13] and implemented in the *RobLoxBioC* package.

## Summary Data Analysis

The most common entry point for the computational biologist is to begin with summarized data obtained from the gene expression module of the BeadStudio/GenomeStudio software. These PC-based programs provide a convenient graphical user interface to import and process BeadArray data from the proprietary format idat files output by Illumina's scanning software. Data are exported from this application in tab-delimited files (separate files for the experimental and control probes) with each row giving the summary information for a particular probe, and different columns for each sample. We recommend exporting raw summary values (which have not been background corrected, transformed, or normalized) at the probe level (“probe profiles”) rather than at the gene level (“gene profiles”) for both regular and control probes to avoid combining probes targeting different transcripts of the same gene in an undesirable manner. Such files can be imported and processed in the R software environment using a range of tools that include *beadarray* [9], *lumi* [14], and *limma* [15].

Another potential source of summarized data are public repositories such as Gene Expression Omnibus (GEO) [16] or ArrayExpress [17]. Experimental data from these databases will generally be summarized and probably normalized, and can be imported into R using the repository-specific packages *GEOquery* [18] and *ArrayExpress* [19].

Once summarized data have been imported into R, quality assessment is necessary to identify poor-quality arrays and check for systematic biases. The *arrayQualityMetrics* [20] package is able to collate quality assessment plots for summarized data created by *beadarray* and identify potential outlier arrays. Boxplots are commonly used to assess the dynamic range from each sample and look for unusual signal distributions (Figure 2C). We also recommend making separate boxplots of regular probes and control probes as a means to highlight unusual samples.

Before comparisons between different biological samples can be made, it is important to remove per-array technical effects to ensure the values being analyzed truly reflect the biology. In the microarray literature, the three steps to achieve this are commonly referred to as background correction (not to be confused with the image processing step of the same name), between-array normalization, and transformation. Two popular methods that implement these steps for Illumina data are neqc and vst from the *limma* and *lumi* packages, respectively.

For background correction, the GenomeStudio option of subtracting the average of the negative controls on an array has been shown on several occasions to be flawed [21]–[23]. One can get by with no background correction and a simple 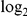 transformation to stabilize variances; however, more sophisticated approaches that use Illumina's negative control probes (sequences with no match to the genome/transcriptome) are preferable. These controls can be used to correct the observed signal intensities from each array using a normal-exponential convolution model [24]–[27] to reduce bias and the number of false positives. Adding a small offset to the corrected intensities has been shown to improve precision and reduce the false discovery rate further. In our research, we routinely use an offset of 16 for neqc to give a good trade-off between variance stabilization and bias. Alternatively, the VST (variance stabilizing transformation) method [28] performs variance stabilization and background correction in the same transformation. Instead of using negative controls, the within-array standard errors calculated from the replicate beads are used to remove the relationship between intensity and signal variability that typically exists.

Negative controls are also useful for estimating the proportion of probes that are expressed in a given sample [29], which can be used to distinguish heterogeneous cell samples from pure samples [29] and to filter out non-expressed probes.

For normalization, between-array quantile is the method most frequently applied to Illumina data both in the literature [21], [27], [30] and in our own research. More sophisticated variants on this approach that use control probes or robust splines (implemented in rsn in *lumi*) have emerged and are increasing in popularity. Strip-level processing, which separates probes depending on physical location and normalizes strips containing the same probes between samples, can also be beneficial for older BeadChip versions [31]. Ultimately, as with other high-throughput technologies, there is no “one-size-fits-all” solution for normalization and the analyst should be prepared to make an informed decision based on exploratory plots and consideration of the assumptions of the method. For instance, classical quantile normalization may be inappropriate in data sets comprising many different tissue types. Standardized data sets and methods of comparison may help guide the analyst in their choice [32].

Next, relationships between a collection of samples can be assessed via multidimensional scaling (MDS, Figure 2D) or principal component analysis (PCA). MDS quantifies sample similarity across many genes (typically the 500 most variable), and reduces the measure to two dimensions for easy viewing. Ideally, samples would separate based on biological variables (sex, treatment, etc.), but often technical effects (such as samples processed together in batches) may dominate the differences between arrays. These effects may be accounted for in a differential expression analysis, or managed using tools such as ComBat [33], [34] or removeBatchEffect within *limma* (as used in Lim et al. (2010) [35]). Employing a good experimental design that ensures biological factors of interest are not confounded with known technical or processing variables is of fundamental importance in any study.

Once data are preprocessed into a normalized “expression matrix” format used throughout Bioconductor, a wide variety of analyses can take place such as clustering, assessing differential expression, classification, and pathway analysis.

## Differential Expression Analysis

Throughout the vignette [6], we make use of the linear modelling framework in the *limma* package for assessing differential expression [36] due to its flexibility and the maturity of the statistical methods it provides. For a designed Illumina experiment, which includes some replication of RNA samples, average log-intensities are estimated for one or more distinct sample types simultaneously using linear models fitted a probe at a time. The *limma* package also allows for observations in a linear model to be weighted according to the confidence in which we hold them. For Illumina BeadArrays, we might naturally want to weight observations by the inverse of the squared standard error (so that observations about which we are more certain are given greater weight), as the standard error should be a function both of the array quality and the number of copies of that type of bead. However, obtaining an accurate measure of the standard error can be problematic. Even if we start out with one, steps such as outlier removal, trimming, background correction at the summary level, and normalization will transform the mean and leave the standard error lacking validity unless it is sympathetically transformed. Thus, it is tempting to assume that our transformation (if we have performed one—e.g., taking logs or using vst) has removed any mean-variance relationship in the data, in which case the number of beads can be used as a weight to account for technical variation that may arise in an experiment. This ignores biological variation between different arrays, and so we should really use a weight consisting of the number of beads contributing to the observation adjusted by an array multiplier that gives a measure of the reliability of the array from which the observation comes. Array-specific weights have been shown to improve power to detect differential expression [37] and are especially useful in human studies where heterogeneity can be high.

Having fitted our weighted linear model, we then set up contrasts between RNA conditions and proceed to estimate between-sample differences of biological interest. Empirical Bayes shrinkage of the probe-wise variances is then applied to ensure that inference is reliable and stable, even when the number of replicate samples is small [36]. These shrunken standard errors are used to calculate moderated *t*-statistics and *F*-statistics (when multiple contrasts are present), and the resulting *p*-values are generally used to rank probes in terms of their evidence for differential expression after adjusting for multiple testing.

## Annotation

By following the steps in the previous section, the researcher may be presented with a list of hundreds if not thousands of differentially expressed probes that are named according to their manufacturer-assigned IDs. At the very least, these must be translated into gene symbols that the researcher can recognize, or into functional pathways that can provide insight into the biological question being investigated.

The Bioconductor project provides infrastructure for mapping between microarray probes and functional genomic annotation to be used in downstream analyses. For Illumina chips, these packages are maintained on a per-organism (e.g., *lumiHumanAll.db*) or per-chip (e.g., *illuminaHumanv3.db*) basis. The organism-specific packages use the nuIDs from Du et al. (2007) [38] to encode the super-set of all probe sequences used in different revisions of chips for the same organism, which can be advantageous when analyzing data from different BeadChip versions. In these packages, the RefSeq IDs provided by Illumina in their own annotation files are used to provide functional annotation for each probe.

However, an important issue that is sometimes taken for granted in the analysis of microarray data is the assignment of genomic and transcriptomic identifiers to each unique probe sequence. Manufacturers provide their own annotation, but inevitably the reported mappings can become outdated as genome or transcriptome versions are updated. This issue was the subject of extensive research for Affymetrix expression arrays (see Dai et al. (2005) [39], amongst others) and has recently been brought to light for Illumina expression [40] and methylation [41] arrays. A significant proportion of probes on each Illumina expression platform are reported to map to non-transcribed genomic regions or have other properties that complicate analyses, such as containing SNPs or repeat-masked elements. Failure to take such factors into account can have a profound effect on the interpretation of microarray data [42]. Barbosa-Morais et al. (2010) [40] describe a scheme to assign a quality score to each probe sequence that captures how well the sequence maps to the genome and transcriptome. Four basic categories, “perfect”, “good”, “bad”, and “no match”, are defined and shown to correlate with expression level and measures of differential expression. We routinely remove probes assigned a bad or no match quality score after normalization. This approach is similar to the common practice of removing lowly expressed probes, but with the additional benefit of discarding probes with a high expression level caused by non-specific hybridization. Besides the obvious benefit of removing probes that are either off-target or promiscuous, such a filtering step reduces the burden of multiple testing and thereby improves the power to detect differential expression. Chip-specific packages such as *illuminaHumanv3.db* and organism-specific packages such as *lumiHumanIDMapping* both provide the user with access to these quality scores.

## Downstream Analyses

There are many other analysis tools available from R/Bioconductor that can be used for downstream analysis of Illumina microarray data. For example, gene ontology/pathway enrichment analysis can be performed with *topGO* or *GOstats* and their associated annotation packages (*GO.db* and *KEGG.db*), as can gene set enrichment analysis using the *GSEAlm* package. In *limma*, both self-contained gene set testing (using the roast function [43]) and competitive gene set testing (using the battery of gene sets available from MSigDB [44]—see the romer function) that operate within the linear model context are possible.

## Conclusions

We have highlighted a number of specially tailored tools and modelling approaches that are available in Bioconductor for the analysis of Illumina gene expression data sets in various formats. A summary of the methods that we currently recommend for Illumina expression analysis are listed in Table 1. Code examples that illustrate how to carry out each of these steps in the analysis are provided in the separate vignette [6] from the *BeadArrayUseCases* package. These Bioconductor tools expand the set of analysis options offered in the vendor-provided GenomeStudio/BeadStudio software, and are continually being developed to accommodate new applications of BeadArray technology, such as methlyation assays.

The open-source Bioconductor platform also presents researchers with a choice of operating system for their analysis and a means to write analysis scripts and generate reports based on them using Sweave [45], which assists with the communication of results and ensures reproducibility of a data analysis. Help is also easy to come by at various levels from manual pages for each function, through to package-specific vignettes and the Bioconductor mailing list for posting questions and reporting problems. Bioconductor software also benefits from a regular release schedule that ensures packages are kept up-to-date with changes in the R software environment [46], which underpins all of this work.
